# Recent advances in breeding and genetics for dairy goats

**DOI:** 10.5713/ajas.19.0381

**Published:** 2019-07-18

**Authors:** Terry A. Gipson

**Affiliations:** 1 American Institute for Goat Research, Langston University, Langston, OK 73050, USA

**Keywords:** Dairy Goats, Breeding, Genetics

## Abstract

Goats (*Capra hircus*) were domesticated during the late Neolithic, approximately 10,500 years ago, and humans exerted minor selection pressure until fairly recently. Probably the largest genetic change occurring over the millennia happened via natural selection and random genetic drift, the latter causing genes to be fixed in small and isolated populations. Recent human-influenced genetic changes have occurred through biometrics and genomics. For the most part, biometrics has concentrated upon the refining of estimates of heritabilities and genetic correlations. Heritabilities are instrumental in the calculation of estimated breeding values and genetic correlations are necessary in the construction of selection indices that account for changes in multiple traits under selection at one time. Early genomic studies focused upon microsatellite markers, which are short tandem repeats of nucleic acids and which are detected using polymerase chain reaction primers flanking the microsatellite. Microsatellite markers have been very important in parentage verification, which can impact genetic progress. Additionally, microsatellite markers have been a useful tool in assessing genetic diversity between and among breeds, which is important in the conservation of minor breeds. Single nucleotide polymorphisms are a new genomic tool that have refined classical BLUP methodology (biometric) to provide more accurate genomic estimated breeding values, provided a large reference population is available.

## INTRODUCTION

Goats (*Capra hircus*) have been modified by humankind ever since their domestication during the late Neolithic, approximately 10,500 years ago, in the Fertile Crescent [1–3]. As domesticated goats spread from the Fertile Crescent, humans exerted minor selection pressure. The largest genetic change happened through natural selection and random genetic drift, the latter causing genes to be fixed in small populations [4]. As humans began to exert selection pressure on domesticated goats, geographical differences arose and those differences have been noted in various studies [5–7]. Two major historical events shaped the raising of goats in Europe, especially northern Europe, and have had an impact on dairy goats worldwide. The first event, which happened approximately 3,000 years after the domestication of the goat, was a small mutation in the human population [8]. This mutation was in a regulatory region near the gene for lactase that allowed lactose tolerance to persist into adulthood [9,10]. With this mutation, goats and cattle could serve another important role other than meat and hides. Milk is a complete food, easily digested, and easily obtained from domesticated, lactating ruminants. This mutation was so important that nearly all Europeans and people of European descent carry this mutation. The second event was the defeat of the Umayyad army by Charles Martel and his Frankish troops near Poitiers (central France) in 732 BCE [11]. One of the items left behind by the hastily retreating Umayyad forces were their goats that they used for milk and cheesemaking. This region of France has become the premier commercial dairy goat hub in the world [12]. With increased selection, regional phenotypic differentiation became more apparent. Interest grew in standardizing animals and later in improving their productivity and one of the early pioneers of breed standardization and improvement was Robert Bakewell, who is considered to be the father of modern animal improvement [13]. From Bakewell’s time on, humans have used many tools to assist in the amelioration of dairy goats. The objective of this paper is to examine those tools. These tools generally include genetic evaluation (biometrics), and most recently genomics.

## BIOMETRICS

Early biometric studies in dairy goats focused upon estimating heritabilities [14–16], which are needed for predicting breeding values, the main component in any genetic evaluation and breed improvement program [17] and upon the estimation of genetic correlations. In addition to the aforementioned genetic studies, projection and adjustment factors specifically for dairy goats were developed [18]. The 305-d lactation, which is the standard lactational length in dairy cattle and by default is the standard lactational length in dairy goats in the United States, was divided into 13 stages, with the shortest stages in early lactation. Sets of factors were developed for four ages at freshening, two seasons, two levels of herd production, and two breed groups [18]. Theses projection factors enabled dairy goat producers to compare objectively records of goats at all stages of lactation. These projected records also enabled more accurate culling and breeding decisions and were a first step towards genetic evaluations of goats, which was to come later. Multiplicative age-season adjustment factors for milk and fat yields of five breeds of dairy goats were also estimated and published [19]. These adjustment factors reflected the changes in milk and fat yields associated with age and season of freshening. As in dairy cattle, these adjustment factors indicated that milk records of goats are affected differently in different seasons for a particular age. Prior to the development of these adjustment factors, goat records were adjusted using age factors for dairy cows [19]. In the early 1980’s, genetic evaluations of production traits of milk and fat yields soon followed and in 1989, genetic evaluation of dairy goats was extended to include evaluation of protein yield and also in 1989, evaluations of milk, fat, and protein yields for Oberhasli and experimental breeds were added [20]. Diverse genetic background of parents was accounted for with an animal model that included all animal relationships. Due to a smaller sample size, the animal model system implemented for dairy goats differed from the one for dairy cattle in that all breeds were processed simultaneously. Genetic trend in 1984 for the five breeds with largest population sizes ranged from 3.8 to 5.2 kg/yr for milk yield. These changes in evaluation procedures improved the usefulness of dairy goat evaluations and aided the dairy goat industry in making genetic improvement [20].

In 1997, type traits were included in the genetic evaluation [21]. Covariance components for final score and 13 linear type traits of dairy goats were estimated by multitrait restricted maximum likelihood using canonical transformation with an animal model. Heritabilities estimates from that study are presented in Table 1. Genetic correlations of linear type traits and final score were positive except for dairyness (−0.15) and teat diameter (−0.10); the largest correlations with final score were 0.66 for fore udder attachment, 0.44 for rear udder arch, 0.36 for rump width, and 0.30 for strength [21]. Later refinements to the animal model were made and genetic progress has continued to chart production and type traits [22,23].

The most recent advancement in the genetic evaluation of production traits in dairy goats has been the incorporation of the test-day model [24]. Test day data for daily milk yield and fat, protein, and lactose content have been analyzed using four test day models with different methodologies to address fixed effects. The reference model contained a fixed effect of year-season of kidding with regression on polynomials nested within the year-season classes, and a random effect of herd test day. In the second model the lactation curve effect from the reference model was replaced by a fixed effect of days in milk (in 3-d periods), the same for all year-seasons of kidding. Two other models were obtained from the reference model by removing the fixed year-season of kidding effect and considering the herd test day effect as either fixed or random, respectively. The models were compared by using two criteria: mean-squared error of prediction and a test of bias affecting the genetic trend. The mean-squared error of prediction indicated a preference for the model with random herd test-day effect, whereas bias preferred the model with the fixed effect of days in milk. Heritability estimates for daily milk yield and milk fat and protein content were 0.26 and 0.24 to 0.27 (Table 1), respectively [24]. Generally, biometric studies have switched from classical studies on heritabilities and genetic correlations of milk and component yields to studies involving longevity [25] or incorporating genomic information into standard genetic evaluations [26].

## GENOMICS

In the beginning of the genomics era in the early 1990’s, research studies investigated the influence of major genes upon productivity traits but has evolved rapidly [27–29]. Marker- or gene-assisted selection has been applied to the α_s1_-casein gene in goats. One study stated that “… the selection for major genes will be more profitable at the breed level if an efficient breeding scheme is already running to be able to account for these optimizations over time [27]”. The knowledge of major genes affecting dairy traits in goats has been well established for protein, and more precisely casein content which is the main component of milk protein. So far, seven variants corresponding to 14 alleles have been identified in many European breeds [30,31] and classified according to their synthesis rate of α_s1_-casein as ‘strong’ (A, B, C), ‘intermediate’ (E), ‘weak’ (F,G) and ‘null’ (O) when α_s1_-casein is absent. The quantitative effects of goat α_s1_-casein variants on dairy traits were studied and have indicated that the difference for protein content between the extreme genotypes AA (favorable) and FF was 4.5 g/kg. These results indicate that milk from AA goats have higher protein, casein, and fat content, and a higher casein: protein ratio than milk from FF goats. A major gene such as α_s1_-casein can also explain a major portion of the total genetic variation in protein content. One study compared estimates of genetic parameters for Alpine goats using an animal model including or excluding α_s1_-casein fixed effect [28]. The heritability of protein content changed from 0.66 to 0.34 when the α_s1_-casein effect was accounted for, showing that variance at this major gene represents about 50% of total genetic variance of protein content in this goat population [28]. When ignoring or accounting for α_s1_-casein, genetic correlations between protein yield and protein content were 0.09 and −0.22, respectively [28,32].

In a recent study in China, a variant within the α_s1_-casein gene (a 11-bp indel variant determined as II, ID, or DD) was related to primiparous litter size in five breeds of Chinese goats. Individuals with the II genotype had the largest litter size when compared with ID or DD genotypes [33]. The authors propose that the 11-bp indel variant (II) could be an effective molecular marker for increasing litter size of goats.

From major genes, the genomic focus shifted to microsatellites for parentage testing [34–36]. Microsatellites, also called short tandem repeats or simple sequence repeats, are scattered throughout the genome of all mammalian species. Microsatellites are identified by constructing polymerase chain reaction (PCR) primers for the DNA flanking the microsatellite region. In one of the original studies on PCR primers, two multiplex systems spanning 11 microsatellite loci each were evaluated for parentage testing in goats [34]. Eight of the loci originated from goats, nine were from cattle and five were from sheep, with 18 of the loci mapped to 16 different autosomes. Parentage exclusion probabilities were computed to be greater than 0.999999 with the probability of finding two identical genotypes is less than 10^−15^. Thus, microsatellite markers are a very reliable tool for parentage identification. The number of PCR primers is periodically evaluated and the number of recommended primer is now 14 (Table 2) [37]. Ancillary to parentage testing, microsatellite markers have been used in pedigree verification [38]. Of the 388 pedigree verifications (dam-offspring) in a nucleus herd of Murciano-Granadina goats in Spain, 71.9% (279) resulted compatible, while 16.2% (63) were incompatible. These errors were probably due to the archaic system used for animal identification and for data transfer. Thus, microsatellite should be considered for verification to reduce errors. This is important because only about a 10% parentage misidentification can reduce genetic progress by as much as 4% [38].

Microsatellites have also been used to evaluate genetic distances among breeds, which is a tool used in the conservation and management of animal genetic resources, especially the conservation of endangered indigenous breeds [39–41]. In a genetic diversity study on nine dairy breeds of Canary Islands goats (Fuerteventura - Feral Esquinzo, Fuerteventura - Feral Ajui-Costa, Fuerteventura - Domestic, Gran Canaria, Lanzarote, La Gomera, La Palma, North Tenerife, and South Tenerife) using 27 microsatellite markers, a mean of 5.9 alleles per population per marker was found and F_IS_ values ranged between 0.45 and −0.48, and F_ST_ was 0.04 [42]. In a structured population, the fixation index, F, is the reduction in heterozygosity relative to Hardy-Weinberg expectation [43]. F can be partitioned into three levels. F_IT_ is the measure of an individual (I) relative to the total population (T), F_IS_ is the measure of an individual (I) relative to the subpopulation (S), and F_ST_ is the measure of a subpopulation (S) relative to the total (T) [43]. F_IS_ (inbreeding coefficient) is interpreted as a correlation measure between alleles rather than a probability, which means that it can have negative values. Positive values of F_IS_ indicate that the individual is less heterozygous than expected given the subpopulation’s heterozygosity, i.e. inbred, and negative values of F_IS_ indicate that the individual is more heterozygous then expected given the subpopulation, i.e. outbred. High F_ST_ (gene fixation coefficient) implies a considerable degree of differentiation among populations. In this Canary Island study, an F_ST_ of only 0.04 indicates that most of the breeds were genetically similar indicating a high degree of interbreeding.

In a study on Chinese dairy goats, the genetic diversity of six breeds, four developed breeds (Guanzhong, Laoshan, Wendeng, and Xinong Saanen) and two introduced breeds (Saanen and Nubian), was examined using 15 microsatellite markers [44]. A mean of 4.9 alleles per population per marker was found, F_IS_ values ranged between 0.09 and −0.08, and F_ST_ was 0.08. The four native Chinese breeds share a common ancestor of Saanen, which was imported from Europe many decades before. There was close genetic relationship between Wendeng and Laoshan and between Guanzhong and Xinong Saanen, which were both in agreement with the formation history and geographical distribution of the breeds in China. As expected, Nubian was genetically distant from Saanen and the four Chinese breeds.

In a Thai dairy goat study on genetic diversity, five imported breeds (Alpine, Jamunapari, Nubian, Saanen, and Toggenburg) were evaluated using five microsatellite markers [45]. A mean of 7.4 alleles per population per marker was found, F_IS_ values ranged between 0.18 and −0.04, and F_ST_ was 0.07. As expected, Alpine, Saanen, and Toggenburg provided one phylogenetic cluster while Jamunapari and Nubian provided two other clusters.

In a Brazilian dairy goat study, Moxotó, which is a local goat breed well-adapted to semiarid Northeast Brazil, was compared to two exotic dairy goat breeds (Alpine and Saanen) using 11 microsatellite markers [46]. Northeast Brazil has the greatest number of goats in the country and imported goat breeds are being crossed with Moxotó, which is eroding the genetic base of the Moxotó. Means of 7.0 alleles for Alpine and Saanen and 3.5 for Moxotó were found, and F_IS_ values ranged between 0.32 and −0.10. F_ST_ was higher for herds (F_ST_ S = 0.08) than for breeds (F_ST_ P = 0.03), indicating similarity between the imported breeds and the existence of crosses between them. As expected, the genetic distance was greatest between Moxotó and the imported breeds, which were of similar phylogenetic clusters.

In addition to the above usages of microsatellite markers, they have been used to verify dairy goat products [47]. Adulteration of cheeses and other dairy goat products can affect the profitability of many small-scale goat producers. An easy method of authentication can contribute to breed sustainability and conservation and improve profitability for dairy goat producers. This could have a significant impact on the rural economy of particular geographic areas with protected status. The Girgentana is an endangered goat breed from Sicily (Italy) and has semi-protected status [48,49]. A panel of 20 microsatellite markers were used on Girgentana, Maltese, and Derivata di Siria goats and eight microsatellite markers alleles were present in Maltese and Derivata di Siria goats but absent in Girgentana goats. Three microsatellite markers (FCB20, SRCRSP5, and TGLA122) were concluded to be the best set of markers, and the authors proposed that these three microsatellite markers could be applied in a breed genetic traceability system of Girgentana dairy products in order to detect adulteration due to Maltese and Derivata di Siria goat breeds.

The successor to microsatellite markers is single nucleotide polymorphisms, frequently called SNPs (pronounced “snips”). A SNP is the single-pair difference at a specific location on specific chromosome. For example, a SNP can occur when the nucleotide cytosine (C) is replaced with the nucleotide thymine (T). SNPs occur normally throughout the genome of all livestock species, including goats. Under the umbrella of the International Goat Genome Consortium (IGGC, http://www.goatgenome.org/), several research projects involved in sequencing the goat genome successfully identified approximately twelve million high quality SNP variants in the goat genome [50]. The goal of the IGGC was to create a SNP database including biological and technical characteristics using reliable bioinformatic SNP detection procedures. In addition, the technological success rate of the SNP design, which included even spacing of SNPs on the genome and selection of minor allele frequencies (MAF) suitable to use in diverse breeds, was an important aspect of IGGC’s SNP database. The SNPs were identified within and between six breeds (Alpine, Boer, Creole, Katjang, Saanen, and Savanna), providing for a robustness for breeds that were even not part of the original study [51]. Validation of the SNP content was conducted with ten goat breeds and 52,295 SNPs were successfully genotyped and used in the manufacturing of a SNP chip (Illumina, Inc, San Diego, CA, USA). The development of the 52K goat SNP chip has accelerated advances in goat genomic studies.

The most obvious starting point for the SNP chip was in evaluating production traits [52,53]. Shortly after the development of the 52K goat SNP chip, a genome-wide association study (GWAS) was conducted on economically important production traits in dairy goats in the UK [53]. A GWAS combines the resulting genotypic data from the SNP chip with phenotypic data such as milk yield [54]. The GWAS on UK dairy goats examined milk yield and udder conformation traits [53]. In that study, the phenotypic data consisted of a total of 137,235 milk yield records on 4,563 goats each scored for 10 conformation traits (udder traits of udder furrow, udder depth, and udder attachment, teat traits of teat shape, teat angle, and teat placement, and feet and leg traits of front legs, back legs, front feet, back feet). The genotypic data consisted of 2,381 of the 4,563 goats genotyped with the 52K goat SNP chip. A genome-wide significant SNP for milk yield was identified on chromosome 19 with additional lesser influential SNPs on chromosomes 4, 8, 14, and 29. Three genome-wide significant SNP for conformation of udder attachment, udder depth, and front legs were identified on chromosome 19 with lesser influential SNPs on chromosomes 4, 5, 6, 10, 11, 12, 13, 14, 15, 16, 17, 18, 21, 23, and 27. However, the proportion of total variance explained by the significant SNPs was low and ranged between 0.4% and 7.0% for milk yield and between 0.1% and 13.8% for conformation traits, thus confirming the polygenic nature of these traits. This conclusion was later supported by a smaller GWAS study on milk yield in U.S. dairy goats [55].

SNPs and GWAS have great potential for traits that are hard or costly to measure [56], and udder health traits are exactly that type of trait because they not only influence milk production but also animal health, milking ability, and longevity. A study in France performed a GWAS on somatic cell count (SCC) as an indirect measure of mastitis resistance [57]. The phenotypic data for the GWAS consisted of SCC on 1,941 Alpine and Saanen goats sired by 20 artificial insemination bucks and the genotypic data of those same females genotyped with the 52K goat SNP chip (Illumina Inc., USA). In the Saanen breed, a genome-wise significant SNP for SCC was identified on chromosome 19, with a region of 33 to 42 Mb in length that included candidate genes associated with response to intramammary infections (retinoic acid receptor α [*RARA*], and Janus kinase signaling pathway genes [*STAT3*, *STAT5A*, and *STAT5B*]). These SNP effects were not present in the Alpine breed. In an Eastern European study using significantly lower animal numbers, researchers found 10 candidate genes (pentraxin 3 [*PTX3*], interleukin-6 [*IL6*], C-type lectin domain family 4 member [*CLEC4E*], interleukin 8 [*IL8*], interleukin 1 receptor antagonist [*IL1RN*], interleukin 15 receptor subunit alpha [*IL15RA*], tumor necrosis factor superfamily member 13 [*TNFSF13*], suppressor of cytokine signaling 3 [*SOCS3*], tumor necrosis factor [*TNF*], and toll-like receptor 3 [*TLR3*]) for resistance to mastitis and gastrointestinal parasitism [58].

Another GWAS study in France examined supernumerary teats (SNT) in goats [59]. Supernumerary teats are undesirable due to impaired machine-milking efficiency, which can increase milking time and lead to possible injury of the udder. The phenotypic data for the GWAS consisted of SNT score (present or absent) on 810 Saanen goats sired by nine artificial insemination bucks and 1,185 Alpine goats sired by 11 artificial insemination bucks and the genotypic data of those same females genotyped with the 52K goat SNP chip (Illumina Inc., USA). No significant SNP effects were found and there was no evidence of segregation of a major gene for SNT in Saanen and Alpine breeds, thus indicating the polygenic nature of this trait.

As with microsatellite marker, a reduced panel SNP chip has been proposed for use in parentage and pedigree verification [60]. In an Italian study, the 52K goat SNP chip (Illumina Inc., USA) was used to develop a 3-step procedure to identify a low-density SNP panel for highly accurate parentage assessment. A reference sample of 109 Alpine goats with known pedigree relationships was genotyped using the 52K chip, then the authors identified a panel of 200 SNPs that was further reduced to two final panels of 130 and 114 SNPs with random coincidental match inclusion (probability of finding two identical genotypes as mentioned earlier in microsatellites) of 1.51×10^−57^ and 2.94×10^−34^, respectively. In the reference data set of 109 Alpine goats, both panels correctly identified all parent–offspring relationships. Further work by these researchers constructed a 195 SNP panel with even greater accuracy [61]. Thus, a reduced panel SNP chip could substitute for microsatellite markers but with a much higher degree of accuracy.

GWAS studies can also identify candidate genes affecting production traits [62]. In a French GWAS, a diacylglycerol O-acyltransferase 1 (*DGAT1*) gene on chromosome 14 was identified as a functional and positional candidate gene affecting fat content. A reference sequence of the *DGAT1* gene was completed and 29 polymorphisms were found, including two novel mutations, R251L and R396W. In the Saanen breed, the frequency of the R251L mutation was 3.5%. In both Saanen and Alpine breeds, the frequency of the R396W mutation was 13% and 7%, respectively. Both mutations were associated with a notable decrease in milk fat content.

## GENOMIC SELECTION

Combining biometrics and genomics yields genomic selection [54,63]. Genomic selection methods yield genomic estimated breeding values (GEBV), which are more accurate than estimated breeding values (EBV) [64]. French researchers are at the forefront in calculating and utilizing GEBV. In France, all Alpine and Saanen progeny-tested bucks were genotyped using the Illumina 52K goat SNP chip. A reference population consisted of 677 bucks and 148 selection candidates. Using a single-step approach with genomic best linear unbiased prediction (GBLUP), prediction accuracy of candidates was improved from 22% to 37% for both breeds compared to the two-step method and was greater than that based on parent average of official evaluations [65]. From that study, the across-breed heritability estimates are presented in Table 1 and are similar to heritability estimates for yield traits that were calculated nearly four decades earlier [14] and type traits calculated nearly two decades earlier [21].

Also, in France, three weighted single-step GBLUP (ssGBLUP) methods were more efficient for detecting SNPs associated with protein content (α_s1_-casein) and yielded a better prediction of genomic breeding values than unweighted ssGBLUP [66]. Unweighted ssGBLUP was also compared to a gene content method that was previously developed to account for multiple α_s1_-casein alleles [67]. However, the gene content method did not improve accuracies of genomic evaluations compared to ssGBLUP probably due the small number of animals genotyped (3,696 Alpine and 3,506 Saanen). The weighted ssGBLUP methods put more weight on SNPs with larger effects, improved accuracies of genomic evaluation over ssGBLUP, and had the added advantage of faster computing, were simpler, and required ssGBLUP to be run only twice.

An English GWAS used single step genomic selection to estimate breeding values (GEBV) for feed efficiency and body weight in a dairy goat herd [68]. Feed accounts for the largest proportion of costs in dairy farming. There are differences in the efficiency of individual animal’s ability to convert energy to milk and the genetic improvement of feed efficiency could reduce feed costs per unit of output. The accuracy of GEBV were low for this population (0.28 for both traits); however, the authors stated that records have only been collected for the last year and the validation population only contained 320 individuals. However, as the reference population containing related animals increases, accuracy is expected to increase also.

A recent study in Spain, using 50,649 lactation records on 19,067 goats of the Florida breed which represented daughters of 4,397 dams and 500 sires, indicated that ssGBLUP methodology increased average reliability of the estimations by 1.06% over classical BLUP methodology [26]. The correlation between A (pedigree relationship) matrix and G matrix was 0.826, which indicated a moderate degree of misidentification of parentage. The correlation between the EBV and GEBV was 0.989 but when only the EBVs of the animals genotyped were compared, the correlation between the estimates obtained with both approaches decreased to 0.952. However, the average reliability of the estimates increased by 5.86%. This increased reliability might be similar to the previous reliability because only 625 animals representing 538 dams and 87 sires were genotyped with the Illumina 52K goat SNP chip.

## CONCLUSION

In the most recent history of the dairy goat, biometric and genomic approaches have been used to select animals of highest genetic merit, and therefore, to improve breeds. Biometric approaches have concentrated upon more accurate estimates of heritabilities and genetic correlations, the former is used in the EBV and the latter is used in the formulation of selection indices. EBV and selection indices have been used in various countries to improve milk, fat, and protein yields. The genomics approach started with microsatellite markers, which have been very important in parentage determination and in genetic conservation studies. Microsatellite markers are an inexpensive means to determine genetic diversity within and across breeds. Most recently, genomics has focused upon SNP and the wealth of information that this technology brings.

## Figures and Tables

**Table 1:** Heritability estimates for production and type traits using a biometrical or genomics approach [citation follows the estimates]

Trait	Biometrical	Genomics
Milk yield (kg)	0.26 [24]	0.30 [65]
Fat (%)	0.24 [24]	0.30 [65]
Protein (%)	0.27 [24]	0.30 [65]
Final score	0.27 [21]	
Stature	0.52 [21]	
Strength	0.29 [21]	
Dairyness	0.24 [21]	
Teat diameter	0.38 [21]	
Rear legs	0.21 [21]	
Rump angle	0.32 [21]	
Rump width	0.27 [21]	
Fore udder attachment	0.25 [21]	0.29 [65]
Rear udder height	0.25 [21]	0.27 [65]
Rear udder arch	0.19 [21]	
Udder depth	0.25 [21]	0.31 [65]
Suspensory ligament	0.33 [21]	0.34 [65]
Teat placement	0.36 [21]	0.29 [65]

**Table 2:** Microsatellites panel primers for parentage testing in goats

Marker	Forward primer sequence (5′-3′)	Reverse primer sequence (5′-3′)	Fragment sizes in bp
CSRD247	GGACTTGCCAGAACTCTGCAAT	CACTGTGGTTTGTATTAGTCAGG	216–240
ILSTS008	GAATCATGGATTTTCTGGGG	TAGCAGTGAGTGAGGTTGGC	174–182
ILSTS19	AGGGACCTCATGTAGAAGC	ACTTTTGGACCCTGTAGTGC	146–152
ILSTS87	AGCAGACATGATGACTCAGC	CTGCCTCTTTTCTTGAGAGC	133–151
INRA005	TTCAGGCATACCCTACACCACATG	AAATATTAGCCAACTGAAAACTGGG	115–121
INRA006	AGGAATATCTGTATCAACCGCAGTC	CTGAGCTGGGGTGGGAGCTATAAATA	107–123
INRA023	GAGTAGAGCTACAAGATAAACTTC	TAACTACAGGGTGTTAGATGAACTC	195–215
INRA063	GACCACAAAGGGATTTGCACAAGC	AAACCACAGAAATGCTTGGAAG	171–177
MAF65	AAAGGCCAGAGTATGCAATTAGGAG	CCACTCCTCCTGAGAATATAACATG	117–135
MCM527	GTCCATTGCCTCAAATCAATTC	AAACCACTTGACTACTCCCCAA	152–164
OARFCB20	GGAAAACCCCCATATATACCTATAC	AAATGTGTTTAAGATTCCATACATGTG	95–105
SRCRSP5	GGACTCTACCAACTGAGCTACAAG	TGAAATGAAGCTAAAGCAATGC	163–179
SRCRSP8	TGCGGTCTGGTTCTGATTTCAC	CCTGCATGAGAAAGTCGATGCTTAG	220–240
SRCRSP23	TGAACGGGTAAAGATGTG	TGTTTTTAATGGCTGAGTAG	77–103

## References

[1] Alberto FJ, Boyer F, Orozco-terWengel P (2018). Convergent genomic signatures of domestication in sheep and
goats. Nat Commun.

[2] MacHugh DE, Bradley DG (2001). Livestock genetic origins: goats buck the trend. Proc Natl Acad Sci USA.

[3] Amills M, Capote J, Tosser-Klopp G (2017). Goat domestication and breeding: a jigsaw of historical,
biological and molecular data with missing pieces. Anim Genet.

[4] Lande R (1976). Natural selection and random genetic drift in phenotypic
evolution. Evolution (N Y).

[5] Canon J, Garcia D, Garcia-Atance MA (2006). Geographical partitioning of goat diversity in Europe and the
Middle East. Anim Genet.

[6] Colli L, Milanesi M, Talenti A (2018). Genome-wide SNP profiling of worldwide goat populations reveals
strong partitioning of diversity and highlights post-domestication migration
routes. Genet Sel Evol.

[7] Oget C, Servin B, Palhière I (2019). Genetic diversity analysis of French goat populations reveals
selective sweeps involved in their differentiation. Anim Genet.

[8] Laland KN, Odling-Smee J, Myles S (2010). How culture shaped the human genome: Bringing genetics and the
human sciences together. Nat Rev Genet.

[9] Sabeti PC, Schaffner SF, Fry B (2006). Positive natural selection in the human lineage. Science.

[10] Fan S, Hansen MEB, Lo Y, Tishkoff SA (2016). Going global by adapting local: A review of recent human
adaptation. Science.

[11] Watson WE (1993). The battle of tours-poitiers revisited. Provid Stud West Civiliz.

[12] Jénot F, Desmaison P (2009). History and strategies of the Charentes-Poitou dairy firms for
goat cheese production: between the logic of the goat sector and the local
area. Renc Rech Rumin.

[13] Gilbert R (2008). From Bakewell to BLUP modern livestock breeding’s short
history.

[14] Iloeje MU, Van Vleck LD, Wiggans GR (1981). Components of variance for milk and fat yields in dairy
goats. J Dairy Sci.

[15] Analla M, Jiménez-Gamero I, Muñoz-Serrano A, Serradilla JM, Falagán A (1996). Estimation of genetic parameters for milk yield and fat and
protein contents of milk from Murciano-Granadina goats. J Dairy Sci.

[16] Iloeje MU, Van Vleck LD (1978). Genetics of dairy goats: a review. J Dairy Sci.

[17] Wiggans GR, Misztal I, Van Vleck LD (1988). Implementation of an Animal Model for genetic evaluation of dairy
cattle in the United States. J Dairy Sci.

[18] Wiggans GR, Van Vleck LD, Dickinson FN (1979). Projection factors for goat lactation records. J Dairy Sci.

[19] Iloeje MU, Rounsaville TR, McDowell RE, Wiggans GR, Van Vleck LD (1980). Age-season adjustment factors for Alphine, LaMancha, Nubian,
Saanen, and Toggenburg dairy goats. J Dairy Sci.

[20] Wiggans GR (1989). Animal model evaluation of dairy goats for milk, fat, and protein
yields with crossbred animals included. J Dairy Sci.

[21] Luo MF, Wiggans GR, Hubbard SM (1997). Variance component estimation and multitrait genetic evaluation
for type traits of dairy goats. J Dairy Sci.

[22] Wiggans GRR, Hubbard SMM (2001). Genetic evaluation of yield and type traits of dairy goats in the
United States. J Dairy Sci.

[23] Manfredi E, Piacere A, Lahaye P, Ducrocq V (2001). Genetic parameters of type appraisal in Saanen and Alpine
goats. Livest Prod Sci.

[24] Andonov S, Ødegård J, Boman IA (2007). Validation of test-day models for genetic evaluation of dairy
goats in Norway. J Dairy Sci.

[25] Castañeda-Bustos VJ, Montaldo HH, Valencia-Posadas M (2016). Linear and nonlinear genetic relationships between type traits
and productive life in US dairy goats. J Dairy Sci.

[26] Molina A, Muñoz E, Díaz C (2018). Goat genomic selection: Impact of the integration of genomic
information in the genetic evaluations of the Spanish Florida
goats. Small Rumin Res.

[27] Barillet F (2007). Genetic improvement for dairy production in sheep and
goats. Small Rumin Res.

[28] Barbieri M, Manfredi E, Elsen J (1995). Effects of the αs1-casein locus on dairy performances and
genetic parameters of Alpine goats. Genet Sel Evol.

[29] Yahyaoui MH, Coll A, Sanchez A, Folch JM (2001). Genetic polymorphism of the caprine kappa casein
gene. J Dairy Res.

[30] Grosclaude F, Mahé M-F, Brignon G, Di Stasio L, Jeunet R (1987). A Mendelian polymorphism underlying quantitative variations of
goat αs1-casein. Genet Sel Evol.

[31] Marletta D, Criscione A, Bordonaro S (2007). Casein polymorphism in goat’s milk. Lait.

[32] Manfredi E, Ricordeau G, Barbieri M, Amigues Y, Bibé B (1995). Genotype at the αs1-casein locus and selection of bucks
on progeny test in the Alpine and Saanen breeds. Genet Sel Evol.

[33] Wang K, Yan H (2018). A novel indel within goat casein alpha S1 gene is significantly
associated with litter size. Gene.

[34] Luikart G, Biju-Duval MP, Ertugrul O, Zagdsuren Y, Maudet C, Taberlet P (1999). Power of 22 microsatellite markers in fluorescent multiplexes for
parentage testing in goats (*Capra hircus*). Anim Genet.

[35] Azhar PM, Chakraborty D, Iqbal Z, Malik AA (2018). Microsatellite markers as a tool for characterization of small
ruminants: a review. Int J Curr Microbiol Appl Sci.

[36] Siwek M, Knol EF (2010). Parental reconstruction in rural goat population with
microsatellite markers. Ital J Anim Sci.

[37] White S, Genestout L, Penedo C (2012). Applied genetics in sheep and goats. ISAG Standing Comm.

[38] Jiménez-Gamero I, Dorado G, Muñoz-Serrano A, Analla M, Alonso-Moraga A (2006). DNA microsatellites to ascertain pedigree-recorded information in
a selecting nucleus of Murciano-Granadina dairy goats. Small Rumin Res.

[39] Pepin L, Amigues Y, Lepingle A, Berthier JL, Bensaid A, Vaiman D (1995). Sequence conservation of microsatellites between Bos taurus
(cattle), Capra hircus (goat) and related species. Examples of use in
parentage testing and phylogeny analysis. Heredity (Edinb).

[40] Ajmone-Marsan P, Colli L, Han JL (2014). The characterization of goat genetic diversity: Towards a genomic
approach. Small Rumin Res.

[41] Dodds KG, McEwan JC, Davis GH (2007). Integration of molecular and quantitative information in sheep
and goat industry breeding programmes. Small Rumin Res.

[42] Martínez AM, Acosta J, Vega-Pla JL (2006). Analysis of the genetic structure of the canary goat populations
using microsatellites. Livest Sci.

[43] Purcell S, Neale B, Todd-Brown K (2007). PLINK: a tool set for whole-genome association and
population-based linkage analyses. Am J Hum Genet.

[44] Wang GZ, Chen SS, Chao TL (2017). Analysis of genetic diversity of chinese dairy goats via
microsatellite markers. J Anim Sci.

[45] Seilsuth S, Seo JH, Kong HS, Jeon GJ (2016). Microsatellite analysis of the genetic diversity and population
structure in dairy goats in Thailand. Asian-Australas J Anim Sci.

[46] Machado TMM, Da Fonseca CG, Rodrigues MT (2006). Genetic diversity between herds of Alpine and Saanen dairy goats
and the naturalized Brazilian Moxotó breed. Genet Mol Biol.

[47] Sardina MT, Tortorici L, Mastrangelo S, Di Gerlando R, Tolone M, Portolano B (2015). Application of microsatellite markers as potential tools for
traceability of Girgentana goat breed dairy products. Food Res Int.

[48] Mastrangelo S, Bonanno A, Simões J, Gutiérrez C (2018). Sustainable Goat Production in Adverse Environments: Volume II.

[49] Mastrangelo S, Tolone M, Montalbano M (2017). Population genetic structure and milk production traits in
Girgentana goat breed. Anim Prod Sci.

[50] Tosser-Klopp G, Bardou P, Bouchez O (2014). Design and characterization of a 52K SNP chip for
goats. PLoS One.

[51] Lashmar SF, Visser C, Van Marle-Köster E, van Marle-Koster E (2015). Validation of the 50k Illumina goat SNP chip in the South African
Angora goat. South African J Anim Sci.

[52] Juditsky A, Nazin A, Tsybakov A, Vayatis N Recursive aggregation of estimators by mirror descent algorithm
with averaging.

[53] Mucha S, Mrode R, Coffey M, Kizilaslan M, Desire S, Conington J (2018). Genome-wide association study of conformation and milk yield in
mixed-breed dairy goats. J Dairy Sci.

[54] Meuwissen T, Hayes B, Goddard M (2016). Genomic selection: a paradigm shift in animal
breeding. Anim Front.

[55] Wasike CB, Rolf M, Silva NCD (2016). Genome-wide association analysis of residual feed intake and milk
yield in dairy goats. J Anim Sci.

[56] Visscher PM, Brown MA, McCarthy MI, Yang J (2012). Five years of GWAS discovery. Am J Hum Genet.

[57] Martin P, Palhière I, Maroteau C (2018). Genome-wide association mapping for type and mammary health
traits in French dairy goats identifies a pleiotropic region on chromosome
19 in the Saanen breed. J Dairy Sci.

[58] Ilie DE, Kusza S, Sauer M, Gavojdian D (2018). Genetic characterization of indigenous goat breeds in Romania and
Hungary with a special focus on genetic resistance to mastitis and
gastrointestinal parasitism based on 40 SNPs. PLoS One.

[59] Martin P, Palhière I, Tosser-Klopp G, Rupp R (2016). Heritability and genome-wide association mapping for
supernumerary teats in French Alpine and Saanen dairy goats. J Dairy Sci.

[60] Talenti A, Nicolazzi EL, Chessa S (2016). A method for single nucleotide polymorphism selection for
parentage assessment in goats. J Dairy Sci.

[61] Talenti A, Palhière I, Tortereau F (2018). Functional SNP panel for parentage assessment and assignment in
worldwide goat breeds. Genet Sel Evol.

[62] Martin P, Palhière I, Maroteau C (2017). A genome scan for milk production traits in dairy goats reveals
two new mutations in *DGAT1* reducing milk fat
content. Sci Rep.

[63] Mucha S, Mrode R, MacLaren-Lee I, Coffey M, Conington J (2015). Estimation of genomic breeding values for milk yield in UK dairy
goats. J Dairy Sci.

[64] Ding X, Zhang Z, Li X (2013). Accuracy of genomic prediction for milk production traits in the
Chinese Holstein population using a reference population consisting of
cows. J Dairy Sci.

[65] Carillier C, Larroque H, Robert-Granié C (2014). Comparison of joint versus purebred genomic evaluation in the
French multi-breed dairy goat population. Genet Sel Evol.

[66] Teissier M, Larroque H, Robert-Granié C (2018). Weighted single-step genomic BLUP improves accuracy of genomic
breeding values for protein content in French dairy goats: A quantitative
trait influenced by a major gene. Genet Sel Evol.

[67] Carillier-Jacquin C, Larroque H, Robert-Granié C (2016). Including αs1 casein gene information in genomic
evaluations of French dairy goats. Genet Sel Evol.

[68] Desire S, Mucha S, Coffey M, Mrode R, Broadbent J, Conington J (2016). Deriving genomic breeding values for feed intake and body weight
in dairy goats. Proceedings of the World Congress on Genetics Applied to Livestock
Production.

